# Complete Genome and Molecular Epidemiological Data Infer the Maintenance of Rabies among Kudu (*Tragelaphus strepsiceros*) in Namibia

**DOI:** 10.1371/journal.pone.0058739

**Published:** 2013-03-20

**Authors:** Terence P. Scott, Melina Fischer, Siegfried Khaiseb, Conrad Freuling, Dirk Höper, Bernd Hoffmann, Wanda Markotter, Thomas Müller, Louis H. Nel

**Affiliations:** 1 Department of Microbiology and Plant Pathology, University of Pretoria, Pretoria, South Africa; 2 Friedrich-Loeffler-Institut, Greifswald-Insel Riems, Germany; 3 Central Veterinary Laboratories, Windhoek, Namibia; Radboud University Medical Centre, NCMLS, The Netherlands

## Abstract

Rabies in kudu is unique to Namibia and two major peaks in the epizootic have occurred since it was first noted in 1977. Due to the large numbers of kudu that were affected, it was suspected that horizontal transmission of rabies occurs among kudu and that rabies was being maintained independently within the Namibian kudu population – separate from canid cycles, despite geographic overlap. In this study, it was our aim to show, through phylogenetic analyses, that rabies was being maintained independently within the Namibian kudu population. We also tested, through complete genome sequencing of four rabies virus isolates from jackal and kudu, whether specific mutations occurred in the virus genome due to host adaptation. We found the separate grouping of all rabies isolates from kudu to those of any other canid species in Namibia, suggesting that rabies was being maintained independently in kudu. Additionally, we noted several mutations unique to isolates from kudu, suggesting that these mutations may be due to the adaptation of rabies to a new host. In conclusion, we show clear evidence that rabies is being maintained independently in the Namibian kudu population – a unique phenomenon with ecological and economic impacts.

## Introduction

Rabies virus (RABV) is typically maintained within species of the mammalian families *Carnivora* and *Chiroptera*. The classical primary host species among carnivores is the dog (*Canis familiaris*), but several other carnivores are known to be able to maintain an independent cycle of RABV, for instance the red fox (*Vulpes vulpes*) in Europe [1] and raccoons (*Procyon lotor*) [2] and skunks (*Mephitis mephitis*) in North America, among others. With these classical trends, the emergence of an extensive rabies cycle in an herbivorous host is unusual. However, in 1977 a rabies epizootic in kudu emerged in Namibia [3] and, with several peaks and troughs in the numbers of observed cases through the decades, is still ongoing today. Due to the geographical extent and the numbers of animals affected, it was suspected that this rabies cycle was maintained within the kudu population.

For rabies, spill-over infections from carnivores to domestic and wild herbivores are known to occur regularly, but such spill-overs are invariably dead-end [4] and serve as an indicator of rabid carnivore (domestic or wildlife) activity [5]. Generally, there are several factors that are necessary for the adaptation and maintenance of rabies in a new host. One factor is the need for overlapping host ranges (sympatry) of a susceptible species with that of a species already maintaining a cycle of RABV [6]. It has also been suggested that the initial infection of a new host is aided by the similarity of the cellular and immunological traits of the new host with the donor, with the number of exposures (due to geographical overlap) being a secondary factor [6]. These factors would explain the relatively high rate of transmission of rabies to new canid hosts (e.g. fox, raccoon, skunk, bat-eared fox etc.) as opposed to non-canid hosts that share the same host range. Another factor that has been suggested is that of recipient host population densities [1], [7].

A limited number of studies have aimed to determine whether specific mutations occur in the RABV genome due to the selective pressures caused by host switching and adaptation. One study noted mutations specific to certain fox species throughout Europe, specifically in the glycoprotein and nucleoprotein genes [1]. However, despite this phylogenetic clustering, different viruses predominantly clustered according to their geographical origin, suggesting that spatial and behavioral patterns of the host species play a greater role in the phylogenetic clustering of RABVs [1], [8]. A second study showed evidence that two types of adaptation could occur: 1) Post-shift adaptation, where the virus mutates in order to better suit the new host; 2) Pre-shift adaptation, in which the virus adapts through convergent evolution to better suit the shift from one host to a new host [9]. It was shown that pre-shift adaptation may have occurred in the introduction of RABVs from bats to skunks and foxes in the Flagstaff region of the USA [9].

As rabies in Namibia frequently affects canids, e.g. dogs and jackals [10], it remained unclear whether rabies cases in kudu are simply a result of a high rate of spillover from these primary hosts, or a truly independent cycle primarily driven by horizontal transmission among these herbivores. Experimental observations of RABV in kudu have suggested that transmission could occur via non-bite means and that the mucosal surfaces of kudu are particularly susceptible to infection [11]. An earlier epidemiological study did consider molecular phylogeny, but included limited sample numbers that did not allow for definitive conclusions about the independence of the kudu rabies cycle [12]. Thus, although the maintenance of an independent RABV cycle in Namibian kudu has been speculated, we intended to provide additional evidence - based on genome sequence properties - that horizontal transmission of rabies among Namibian kudu is indeed a common occurrence. Such evidence included new phylogenetic data and the identification of unique amino acid (aa) changes in viruses recovered from kudu, as opposed to those from canid host species.

## Methods

### Partial N gene sequencing

**Figure 1:**
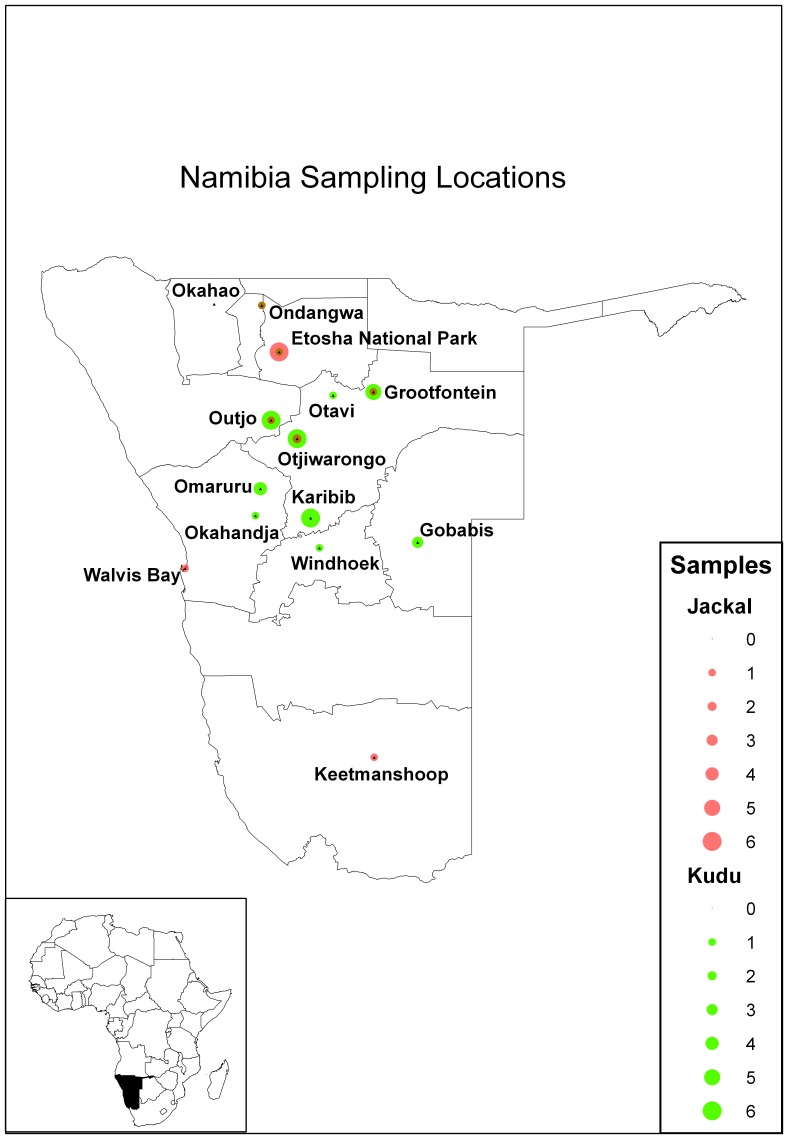
Kudu and jackal sample origins. Numbers and locations of all rabies virus samples used in the partial sequencing analysis of this study from kudu (green) and jackal (red). The size of the dot increases with the number of samples from each location.

Brain samples used in this study were received from the Central Veterinary Laboratory (CVL) in Windhoek, Namibia. The brain samples were obtained from dead, or suspected rabid animals. These samples were from kudu and jackal - from years 2008 and 2009 - that tested positive for rabies after being submitted for diagnostic testing (Table 1) via a network of farmers, interest groups and wildlife conservancies that was established for this study in Namibia. All samples obtained through this network were used in the study. These samples were from various regions throughout Namibia (Figure 1), although the majority originated from central Namibia where the game farming industry is predominant. In total, 49 fluorescent antibody test (FAT) positive samples were sequenced. RNA extraction was performed using Trizol (Invitrogen) according to the manufacturer's instructions. The RT-PCR reaction targeted a region (bases 16-646 according to the Pasteur virus rabies genome, GenBank accession number: M13215) of the nucleoprotein gene approximately 602 bp in size [13]. PCR amplicons were first confirmed by agarose gel electrophoresis, followed by PCR purification using the Wizard® SV Gel and PCR Clean-Up System (Promega), according to the manufacturer's instructions.

**Table 1:** Brain samples from various species used in the phylogenetic analysis of partial RABV nucleoprotein gene sequences.

Sample Number	GenBank Number	Species#	Year of Isolation	Origin - District	Origin - Region	Country	Reference
3K08	JQ691415	Kudu	2008	Okahandja	Otjozondjupa	Namibia	This study
8K08	JQ691416	Kudu	2008	Okahandja	Otjozondjupa	Namibia	This study
18K08	JQ691417	Kudu	2008	Omaruru	Erongo	Namibia	This study
19K08	JQ691418	Kudu	2008	Gobabis	Omaheke	Namibia	This study
24K08	JQ691420	Kudu	2008	Outjo	Kunene	Namibia	This study
27K08	JQ691421	Kudu	2008	Outjo	Kunene	Namibia	This study
28K08	JQ691422	Kudu	2008	Outjo	Kunene	Namibia	This study
33K08	JQ691423	Kudu	2008	Grootfontein	Otjozondjupa	Namibia	This study
35K08	JQ691424	Kudu	2008	Grootfontein	Otjozondjupa	Namibia	This study
46K08	JQ691426	Kudu	2008	Outjo	Kunene	Namibia	This study
51K08	JQ691427	Kudu	2008	Okahandja	Otjozondjupa	Namibia	This study
52K08	JQ691428	Kudu	2008	Okahandja	Otjozondjupa	Namibia	This study
55K08	JQ691429	Kudu	2008	Gobabis	Omaheke	Namibia	This study
57K08	JQ691430	Kudu	2008	Otjiwarongo	Otjozondjupa	Namibia	This study
59K08	JQ691431	Kudu	2008	Okahandja	Otjozondjupa	Namibia	This study
60K08	JQ691432	Kudu	2008	Outjo	Kunene	Namibia	This study
95K08	JQ691437	Kudu	2008	Omaruru	Erongo	Namibia	This study
130K09	JQ691438	Kudu	2009	Outjo	Kunene	Namibia	This study
131K09	JQ691439	Kudu	2009	Otjiwarongo	Otjozondjupa	Namibia	This study
142K09	JQ691440	Kudu	2009	Otjiwarongo	Otjozondjupa	Namibia	This study
143K09	JQ691441	Kudu	2009	Windhoek	Khomas	Namibia	This study
144K09	JQ691442	Kudu	2009	Gobabis	Omaheke	Namibia	This study
146K09	JQ691443	Kudu	2009	Omaruru	Erongo	Namibia	This study
147K09	JQ691444	Kudu	2009	Otjiwarongo	Otjozondjupa	Namibia	This study
152K09	JQ691446	Kudu	2009	Okahandja	Otjozondjupa	Namibia	This study
153K09	JQ691447	Kudu	2009	Grootfontein	Otjozondjupa	Namibia	This study
158K09	JQ691448	Kudu	2009	Otjiwarongo	Otjozondjupa	Namibia	This study
172K09	JQ691449	Kudu	2009	Etosha National Park	Kunene	Namibia	This study
190K09	JQ691452	Kudu	2009	Grootfontein	Otjozondjupa	Namibia	This study
191K09	JQ691453	Kudu	2009	Otjiwarongo	Otjozondjupa	Namibia	This study
201K09	JQ691457	Kudu	2009	Omaruru	Erongo	Namibia	This study
212K09	JQ691459	Kudu	2009	Karibib	Erongo	Namibia	This study
234K09	JQ691460	Kudu	2009	Ondangwa	Ohangwena	Namibia	This study
240K09	JQ691462	Kudu	2009	Grootfontein	Otjozondjupa	Namibia	This study
244K09	JQ691463	Kudu	2009	Otavi	Otjozondjupa	Namibia	This study
20J08	JQ691419	Jackal	2008	Okahao	Omusati	Namibia	This study
38J08	JQ691425	Jackal	2008	Grootfontein	Otjozondjupa	Namibia	This study
64J08	JQ691433	Jackal	2008	Keetmanshoop	Karas	Namibia	This study
67J08	JQ691434	Jackal	2008	Etosha National Park	Kunene	Namibia	This study
89J08	JQ691435	Jackal	2008	Etosha National Park	Kunene	Namibia	This study
93J08	JQ691436	Jackal	2008	Otjiwarongo	Otjozondjupa	Namibia	This study
151J09	JQ691445	Jackal	2009	Walvis Bay	Erongo	Namibia	This study
178J09	JQ691450	Jackal	2009	Etosha National Park	Kunene	Namibia	This study
179J09	JQ691451	Jackal	2009	Etosha National Park	Kunene	Namibia	This study
192J09	JQ691454	Jackal	2009	Etosha National Park	Kunene	Namibia	This study
193J09	JQ691455	Jackal	2009	Etosha National Park	Kunene	Namibia	This study
197J09	JQ691456	Jackal	2009	Outjo	Kunene	Namibia	This study
204J09	JQ691458	Jackal	2009	Otjiwarongo	Otjozondjupa	Namibia	This study
236J09	JQ691461	Jackal	2009	Ondangwa	Ohangwena	Namibia	This study
RV385	AY330733	Jackal	1988	Ghanzi	Ghanzi	Botswana	[16]
RV389	AY330737	Jackal	1990	Orapa	Central	Botswana	[16]
RV444	AY330749	Dog	1991	Maun	Ngamiland	Botswana	[16]
RV447	AY330752	Dog	1991	Maun	Ngamiland	Botswana	[16]
RV481	AY330761	Jackal	1991	Orapa	Central	Botswana	[16]
RV1487	DQ194855	Kudu	2003	Windhoek	Khomas	Namibia	[12]
RV1488	DQ194856	Kudu	2003	Windhoek	Khomas	Namibia	[12]
RV1489	DQ194857	Kudu	2003	Omaruru	Erongo	Namibia	[12]
RV1490	DQ194858	Kudu	2003	Okahandja	Otjozondjupa	Namibia	[12]
RV1491	DQ194859	Kudu	2003	Omaruru	Erongo	Namibia	[12]
RV1492	DQ194860	Kudu	2003	Okahandja	Otjozondjupa	Namibia	[12]
RV1493	DQ194861	Kudu	2003	Okahandja	Otjozondjupa	Namibia	[12]
RV1494	DQ194862	Kudu	2003	Omaruru	Erongo	Namibia	[12]
RV1498	DQ194865	Jackal	2000	Khorixas	Kunene	Namibia	[12]
RV1504	DQ194871	Jackal	2003	Windhoek	Khomas	Namibia	[12]
RV1508	DQ194875	Jackal	2003	Windhoek	Khomas	Namibia	[12]
RV1510	DQ194877	Dog	2000	NK	Oshikoto	Namibia	[12]
RV1511	DQ194878	Dog	2000	Oshakati	Oshana	Namibia	[12]
RV1514	DQ194880	Dog	2003	Rundu	Kavango	Namibia	[12]
RV1517	DQ194882	Dog	2003	Okahandja	Otjozondjupa	Namibia	[12]
RV1518	DQ194883	Eland	2003	Omaruru	Erongo	Namibia	[12]
RV1825	DQ489835	Bat-eared fox	1990	Gordonia	Northern Cape	South Africa	[17]
RV1826	DQ194885	Bat-eared fox	NK	Etosha	Kunene	Namibia	[17]
RV1827	DQ194886	Bat-eared fox	NK	Etosha	Kunene	Namibia	[12]
RV1828	DQ489836	Bat-eared fox	NK	Etosha	Kunene	Namibia	[17]
RV1829	DQ194887	Jackal	NK	Etosha	Kunene	Namibia	[17]
RV1830	DQ194888	Dog	NK	NK	NK	Namibia	[12]
RV1831	DQ489837	Bat-eared fox	1990	Postmasburg	Northern Cape	South Africa	[17]
RV1857	DQ489853	Bat-eared fox	1994	Namaqualand	Northern Cape	South Africa	[17]
RAVMMGN	M13215	Pasteur virus					[21]

NK - unknown information.

# Kudu – *Tragelaphus strepsiceros*; Dog – *Canis familiaris*; Bat-eared fox – *Otocyon megalotis*; Jackal – *Canis mesomelas*; Eland – *Tragelaphus oryx*.

Purified PCR products were sequenced using the BigDye Terminator v3.1 Kit cycle sequencing protocol (Applied Biosystems) at the University of Pretoria on an ABI3300 DNA Sequencer. Sequences were edited using CLC Main Workbench v6.0 (CLCBio). For phylogenetic analysis, the newly obtained sequences - as well as various relevant sequences from previous studies (Table 1) - were aligned using the Clustal X function in BioEdit [14]. A Neighbour-joining phylogenetic tree was constructed with the use of the Kimura 2-parameter model with 1000 bootstrap replications in MEGA 5.0 [15].

### Full genome sequencing

#### RNA extraction

RNA was extracted from two kudu and two jackal brain samples (Table 2) using a protocol combining Trizol (Invitrogen) and RNeasy mini kit (Qiagen) according to the manufacturer's instructions.

**Table 2:** Full genome RABV sequences from kudu and jackals constructed in this study.

Sample Number	GenBank accession no.	Species#	Year of Isolation	Origin – District	Origin - Region	Country
239K09	JX473840	Kudu	2009	Windhoek	Khomas	Namibia
240K09	JX473841	Kudu	2009	Grootfontein	Otjozondjupa	Namibia
178J09	JX473838	Jackal	2009	Etosha National Park	Kunene	Namibia
192J09	JX473839	Jackal	2009	Etosha National Park	Kunene	Namibia

# Kudu – *Tragelaphus strepsiceros*; Jackal – *Canis mesomelas.*

#### Reverse-Transcription Polymerase Chain Reaction (RT-PCR)

A One-Step Reverse Transcription PCR was performed using the SuperScript® III One-Step RT-PCR kit (Invitrogen) for products less than 1500 base pairs in length, according to the manufacturer's instructions with several primer pairs (Table S1). The following cycle conditions were used on a model 2720 thermocycler (Applied Biosystems): 50°C for 30 minutes; 95°C for 2 minutes and 42 cycles of 95°C for 30 seconds, 55°C for 30 seconds, 68°C for 60 seconds; and a final extension step for 5 minutes at 68°C.

A long range One-Step RT-PCR was performed using the SuperScript® One-Step RT-PCR for Long Templates (Invitrogen), according to the manufacturer's instructions with the following conditions: 50°C for 30 minutes, 95°C for 2 minutes followed by 42 cycles of 95°C for 30 seconds, 55°C for 30 seconds, 68°C for 1 minutes/kb. A final extension step was performed for 5 minutes at 68°C.

PCR products of sizes less than 1500 bp were analysed by 1.5% agarose gel electrophoresis using 1× TAE buffer (1.6 M Tris-acetate, 40 mM EDTA). For products larger than 1500 bp, a 1% agarose gel was prepared. A 100 bp DNA molecular weight marker (Promega) was included to identify the size of the amplicons.

#### Purification and sequencing

PCR amplicons generated were purified using the QIAquick Gel Extraction Kit (Qiagen), according to the manufacturer's instructions. PCR products were sequenced using the BigDye Terminator v1.1 Kit cycle sequencing protocol (Applied Biosystems) with slight modifications. One microlitre 5× sequencing buffer, 1µl 5 pmol/µl primer (Table S1), 5µl [15 ng/µl] template, 1µl nuclease free molecular grade water (Qiagen) and 2µl BigDye Terminator mix v1.1 was added to a final volume of 10 µl. The reaction was performed using the following profile: an initial denaturation step at 96°C for 1 min; then 26 cycles of 96°C for 15 seconds; 53°C for 10 seconds; and 60°C for 4 minutes.

Following the sequencing reaction, sequence products were purified using Sigma Spin Sequencing Reaction Clean-up Post Reaction Purification Columns (Sigma) according to the manufacturer's instructions. Fifteen microlitres of the eluate were added to 15μl of Hi-Di-Formamide and subsequently analysed using an ABI 3130 DNA sequencer (Applied Biosystems).

#### Genetic analyses

Sequences were assembled, translated and annotated using CLC Main Workbench v6 (CLCBio) and the ClustalX function of BioEdit [14]. Multiple alignments including sequences from the public domain (Table 3) were then manually screened for nucleotide sequence differences and those that also resulted in aa changes were identified. To identify any known viruses with the same aa in the variable positions identified, we used a blast analysis, screening the protein data bases in Genbank with 7 aa sequence segments that were composed of the variable aa and the 3 aa immediately to the left and right of this position. Finally, phylogenetic analysis was performed with the inclusion of the full genome sequences presented in Table S2 and according to the methodology described for partial N gene sequences, above.

**Table 3:** Lyssavirus isolates used in comparative amino acid analyses.

Virus isolate	Country and area of isolation	Host species*	Year of isolation	Laboratory reference number	GenBank accession numbers
RABV (canid variant)	Sibasa, South Africa	Dog	2006	262/06	HM179504 (N), HQ266628 (P), HQ266609 (M), HQ266620 (G).
RABV (canid variant)	emKhondo, formerly Piet Retief, South Africa	Dog	2004	567/04	HM179505 (N), HQ266626 (P), HQ266607 (M), HQ266618 (G).
RABV (canid variant)	Thabazimbi, South Africa	Dog	1996	479/96	HM179506 (N), HQ266625 (P), HQ266610 (M), HQ266621 (G).
RABV (canid variant)	Soutpansberg, South Africa	Black-backed jackal	2005	819/05	HM179507 (N), HQ266629 (P), HQ266611 (M), HQ266622 (G).
					
RABV (canid variant)	Umtata, South Africa	Bat-eared fox	2005	31/05	HM179508 (N), HQ266627 (P), HQ266608 (M), HQ266619 (G).
RABV (canid variant)	Japan	Laboratory strain	1915	Nishigihara	AB044824
RABV (canid variant)		SAD strain derivative		ERA	EF206707
RABV (canid variant)	USA	Silver-haired bat	1983	SBBRV-18	AY705373
RABV (canid variant)	Japan	Nishigahara derivative	1918	RC-HL	AB009663
RABV (canid variant)	China	Human		Flury-LEP	FJ577895
RABV (canid variant)	USA	LEP-Fury derivative	1939	HEP-Flury	AB085828
RABV (canid variant)		Vaccine		PV	M13215
RABV (mongoose variant)	Rusape, Zimbabwe	Slender mongoose	1994	22107	FJ392391 (N), HQ266633 (P), HQ266615 (M), FJ465408 (G).
RABV (mongoose variant)	Grootgewaagd, South Africa	Yellow mongoose	1990	669/90	FJ392385 (N), HQ266616 (M), FJ465402 (G).
RABV (mongoose variant)	Kroonstad, South Africa	Yellow mongoose	1995	767/95	FJ392388 (N), HQ266630 (P), HQ266617 (M), FJ465405 (G).
RABV (mongoose variant)	Uitenhage, South Africa	Yellow mongoose	1996	364/96	FJ392379 (N), HQ266632 (P), HQ266614 (M), FJ465397 (G).
RABV (mongoose variant)	Beaufort West, South Africa	Water mongoose	1991	113/91	FJ392372 (N), HQ266631 (P), HQ266613 (M), FJ465390 (G).
					
LBV	Durban, South Africa	Wahlbergs Epauletted Fruit bat	2008	LBVSA2008	HM179509 (N), HQ266634 (P), HQ266612 (M), HQ266623 (G).
					
LBV	Amanzimtoti, South Africa	Wahlbergs Epauletted Fruit bat	2006	LBVSA2006	EF547452 (N), EF547414 (P), EF547435 (M), EF547422 (G).
					
LBV	Exported to France from an unknown African origin	Egyptian fruit bat	1999	LBVAFR1999	EF547447 (N), EF547418 (P), EF547445 (M), EF547432 (G).
					
LBV	Lagos Island, Nigeria	Straw-coloured fruit bat	1956	LBVNIG1956	EF547459 (N), EF547407 (P), EF547444 (M), EF547431 (G).
					
LBV	Durban, South Africa	Wahlbergs Epauletted Fruit bat	2004	LagSA2004	EF547458 (N), EF547415 (P), EF547440 (M), EF547428 (G).
					
LBV	Westville, South Africa	Slender mongoose	2004	Mongoose2004	EF547453 (N), EF547409 (P), EF547438 (M), EF547423 (G).
					
MOKV	Bulawayo, Zimbabwe	Cat	1981	12341	FJ465417 (N), GQ861350 (P), GQ472991 (M), GQ473003 (G).
					
MOKV	East London, South Africa	Cat	1995	543/95	FJ465415 (N), GQ500116 (P), GQ472992 (M), GQ500110 (G).
MOKV	Pinetown, South Africa	Cat	1997	252/97	Unpublished (N) AF369376 (P), GQ472997 (M), GQ500112 (G).
MOKV	East London, South Africa	Dog	2006	173/06	FJ465412 (N), GQ861351 (P), GQ472999 (M), HQ266624 (G).
					
MOKV	Zimbabwe	Cat	1981		S59447
DUVV	Pilanesberg, South Africa	Human	2006	DUVVSA2006	EU623444
DUVV	Louis Trichardt, South Africa	Insectivorous bat (Unidentified)	1981	DUVVSA1981	EU623438 (N), EU623439 (P), EU623441 (M), EU623443 (G).
					
DUVV	Bela Bela, South Africa	Human	1970	DUVVSA1970	EU623437 (N), EU623436 (P), EU623440 (M), EU623442 (G).
					

* Dog – *Canis familiaris*; Black-backed jackal – *Canis mesomelas*; Bat-eared fox – *Otocyon megalotis*; Human – *Homo sapiens sapiens*; Cat – *Felis domesticus*; Slender mongoose - *Galerella sanguinea*; Water mongoose - *Atilax paludinossus*; Yellow mongoose - *Cynictis penicillata*; Silver-haired bat - *Lasionycteris noctivagans*; *Egyptian fruit bat – Rousettus aegyptiacus*; Wahlbergs Epauletted Fruit bat - *Epomophorus wahlbergi*; Straw-coloured fruit bat - *Eidolon helvum*.

#### Rapid Amplification of cDNA Ends (RACE)

Rapid Amplification of cDNA Ends (RACE) determined the 5′- and 3′ -termini of viral RNA from samples 239K09, 240K09, 178J09 and 192J09. RACE was performed with freshly extracted RNA using the 5′- and the 3′-RACE system from Invitrogen (Invitrogen, Carlsbad, USA) according to the manufacturer's recommendations. Due to a lack of a poly-A tail at the 3′-terminus of the viral RNA, a poly-C tailing was done prior to 3′-RACE. Some modifications were introduced to the 3′-RACE procedure because of the poly-C tailing: for first strand cDNA synthesis, amplification of the cDNA and subsequent hemi-nested PCR of the abridged anchor primer (AAP) from the 5′ RACE kit had to be used instead of the recommended adapter primer (AP, designed for annealing to a poly-A tail). As gene specific primers (GSPs) for the 3′-RACE system RAB-JACKAL-150R (amplification of cDNA) and RAB-JACKAL-100R (hemi-nested PCR) and for the 5′-RACE procedure RAB-JACKAL-12235F (cDNA synthesis) and RAB-JACKAL-12265F (amplification of cDNA) were used for all four samples (Table S1). Amplification products were visualized on a 2% agarose gel, purified and subsequently sequenced (see Purification and sequencing).

## Results

### Phylogenetic analyses

**Figure 2:**
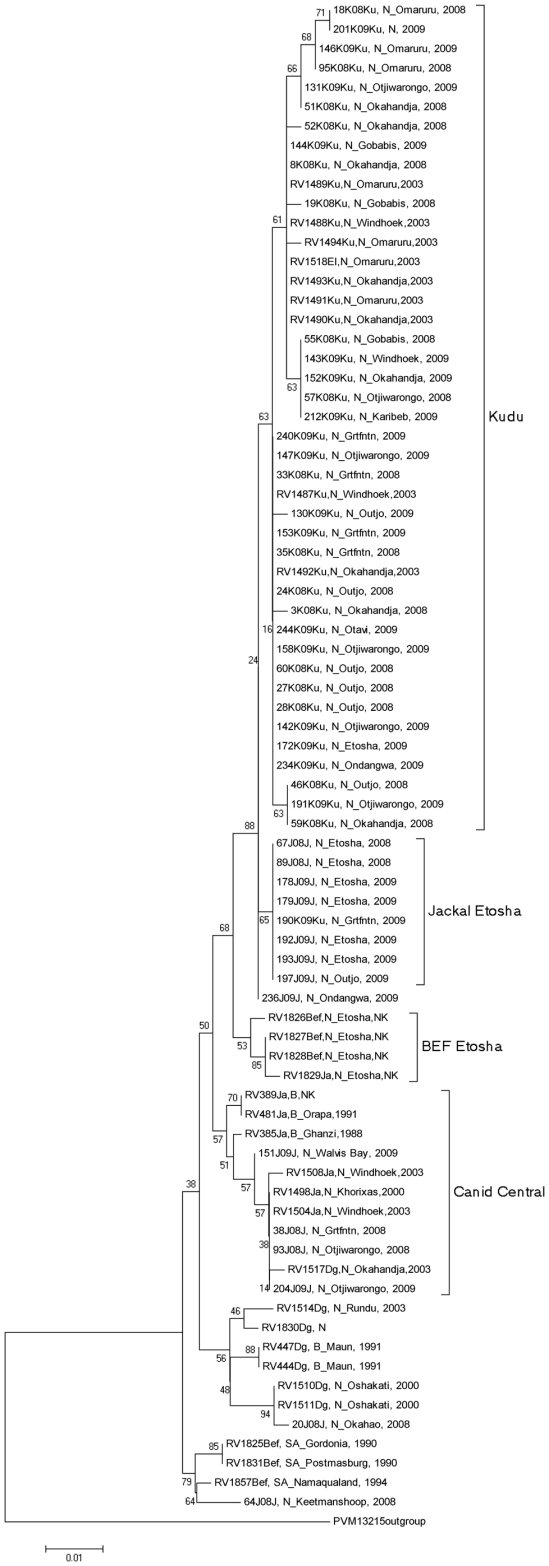
Partial N gene phylogenetic analysis. Neighbour-joining phylogenetic tree with 1000 bootstrap replications of partial RABV nucleoprotein gene sequences generated in this study as well as representative sequences from South Africa and Namibia. Pasteur virus was used as an outgroup. Samples labeled as follows: Isolate number, species, country, region, and year. Ku = kudu; J = Jackal; Dg = Dog; Bef = Bat-eared fox; El = Eland; SA = South Africa; N = Namibia; B = Botswana; Grtfntn = Grootfontein; NK = Not known.

A few previous studies have included partial nucleoprotein gene sequencing of Namibian and other southern African RABV isolates and a sizeable collection of sequences can be found in Genbank [12], [16], [17]. We therefore chose to continue with this sequence region in our analyses of the 49 new isolates from kudu and jackal (35 kudu and 14 jackal) (Table 1) from regions throughout Namibia (Figure 1). Subsequent phylogenetic analysis depicted that 42 from 43 kudu RABV isolates belonged to a single clade that were distinct from any other RABV isolates from Namibia, or elsewhere in southern Africa (Figure 2). The single exception was the kudu specimen 190K09 from Grootfontein, which bundled with a group consisting of 7 jackal isolates from Etosha National Park. The majority of RABV from jackals sequenced were from Etosha National Park (7/14) and these all grouped together; separate from a rabies cycle in bat-eared foxes – also from the Etosha National Park (Figure 2). Samples 151J09 and 204J09 grouped with jackal and dog RABV sequences from Botswana and central Namibia. Sample 64J08, originating from Keetmanshoop in southern Namibia, grouped closely with other RABV sequences from a South African bat-eared fox rabies cycle from areas bordering Namibia to the south. One eland (*Tragelaphus oryx*) sample from a previous study (RV1518) also grouped with the RABV sequences from kudu.

**Figure 3:**
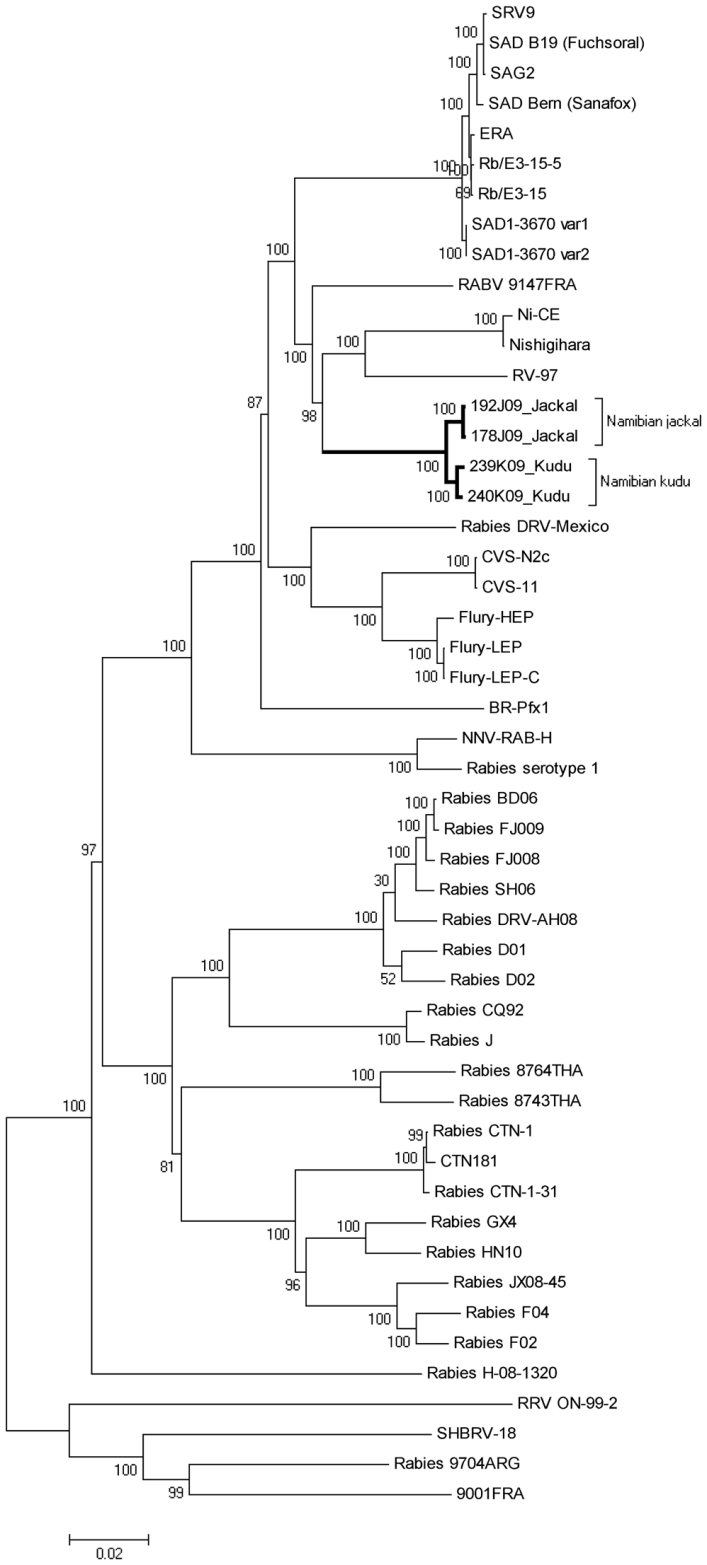
Complete genome phylogenetic analysis. Neighbour-joining phylogenetic tree with 1000 bootstrap replicates, constructed using the Kimura 2-parameter model, of RABV full genomes sequenced in this study (bold branches) as well as RABV full genomes available on GenBank.

We also wanted to compare and evaluate phylogeny based on full genome sequences, where the resultant tree (Figure 3) supported the results obtained from the partial N gene sequences shown above. The 4 isolates that were sequenced in full grouped separately from any other known sequences, and the 2 isolates from jackal (178J09 and 192J09) grouped separately from the 2 isolates from kudu (239K09 and 240K09) with 100% bootstrap support. The next closest strains were both vaccine strains from Russia and Japan respectively. The separate grouping of the viruses sequenced in this study was expected as no other full genome sequences of African RABV isolates were available, and the distinction between kudu and jackal isolates was consistent with a hypothetical genetic drift between jackal and kudu rabies cycles.

### Amino acid sequence analysis of full genomes

The full genomes of the 2 RABV isolates from jackal were found to be identical with respect to aa sequence for all five genes, while the 2 isolates from kudu differed from one another in only one aa position, in the L gene. However, there were 13 aa differences between the kudu and the jackal RABV isolates. Three aa variations in the G gene were unique to the kudu RABV isolates and a further variation unique to both kudu and jackal RABV (L gene), even after comparing these aa to all other lyssavirus G and L sequences known (non-redundant protein and Swiss-Prot databases, Table 4).

**Table 4:** Amino acid variations unique to kudu and jackal RABV genomes when compared to all known lyssavirus sequences.

Gene	Host species	Position	Variable amino acid	Amino acid in other RABV (Table 3)	Charge change
Glycoprotein	Kudu	34	Asparagine	Serine	none
Glycoprotein	Kudu	112	Threonine	Alanine	non-polar to polar
Glycoprotein	Kudu	191	Serine	Asparagine	none
Polymerase	Kudu and Jackal	1140	Serine	Glycine	non-polar to polar

## Discussion

RABV is typically maintained within carnivorous hosts and maintenance within an herbivorous host population has never been proven. The apparently independent cycling of a RABV variant within Namibian kudu, known to be a non-aggressive herbivore, is therefore truly unique. In this study, we aimed to determine whether the RABVs isolated from Namibian kudu showed characteristics of host adaptation and whether they represented a distinct cycle of the disease. In general, phylogenetic analyses have shown clustering of RABVs to occur according to the geographical origin of the viruses, despite the fact that the viruses were isolated from several different species [1]. However, some exceptions have been noted, more specifically and predominantly, in bat species in the Americas [18]. Using molecular analyses, we also intended to determine whether unique mutations had occurred in the cross-species transmission from carnivores to kudu in order to: 1) further support/reject the notion of an independent cycle and; 2) determine whether the particular susceptibility of kudu to RABV infection is due to the adaptation of the virus to a new host. Historical epidemiological data is clear on the directionality of original transmission pathways for rabies in Namibia. Endemic cycles in jackal were firmly established at the turn of the 20^th^ century, with the first recorded cases in kudu appearing several decades later [5], [7]. With the advent of molecular genetics, the close relationship between the viruses in jackal and kudu was shown – and the establishment of an independent kudu cycle is demonstrated in the present study.

Phylogenetic analyses of partial N gene sequences from 35 kudu and 14 jackal RABVs showed a significant clustering of all of the sequences from kudu RABVs separately from any other sequences from canid RABVs, with high bootstrap confidence. As the samples were all taken within the same temporal and similar spatial range, the divergence seen between RABV from canids and kudu can only be explained by the existence of a RABV cycle that is being maintained within the kudu population, separately to other RABV cycles in canid populations. An exception to the general clustering of RABV from kudu is sample 190K09, which grouped with RABV from jackals from Etosha National Park. This can be explained by a spill-over infection from a jackal to that kudu, as is commonly seen in cases of bovine infections from rabid jackals [10], [19]. It is a foregone conclusion that rabies was first introduced into the kudu population in this manner and that sporadic subsequent spillovers could be expected, but not on a scale that would account for the numbers of kudu rabies cases recorded since 1977. In addition, if a separate rabies cycle was not being maintained within the kudu population, several RABVs from canids would be interspersed within the kudu group with the assumption that one jackal could infect several kudu. It was also noted that several samples from jackals from the central regions of Namibia (where the majority of samples from kudu were taken), grouped in separate RABV cycles with other RABVs from jackals and dogs from the central region (Figure 2). This lends further support to the observation that RABV is being maintained in a separate cycle in kudu.

Sequence analyses revealed several aa variations unique to Namibian kudu RABVs. The variations specifically seen in the RABV isolates from kudu may suggest that these changes arose due to the adaptation of the virus to the host, but this notion will remain speculative until proven. Alternatively, this may suggest the divergence of the virus due to a separate RABV cycle being maintained within the kudu population (geographical and temporal drift), as kudu in Namibia may be geographically isolated due to separation of these animals by barriers such as game fences. Variations observed in viruses from both kudu and jackal may be due to the geographical isolation (on a larger scale) of the viruses from other rabies viruses and RABV cycles in Namibia and southern Africa, and support the evolutionary link between kudu and jackal rabies. In contrast, a recent study showed that RABVs did not undergo significant mutational differences after the host shift from bats to skunks, in both the coding and non-coding regions of the genomes [9]. However, the epizootic and maintenance of rabies in skunks occurred in a brief time frame in comparison with the emergence of rabies in kudu, and thus the time for divergence is greater in the kudu RABV cycle.

Complete genome sequencing is an important and useful method utilized in this study. The need for more complete genome sequences is becoming more and more important, especially with the molecular data that can only be generated through this technique. This study has shown the need for more complete genome sequencing as the sequences from this study were the first complete genome sequences for RABV from sub-Saharan Africa. These sequences will be important for a future databank of complete genomes that can be used for improved phylogenetic resolution as well as other molecular studies. Further work will need to be performed in order to determine virus evolution, the effects of certain mutations on protein folding, as well as continued insight into host-virus interactions and pathogenicity [20].

In conclusion, phylogenetic and full genome sequence analyses showed several correlations between Namibian ‘street viruses’ to attenuated vaccine strains, which can be explained by the introduction of the cosmopolitan RABV strain from Europe into Africa during the mid-20^th^ century. Most importantly, several unique mutations were observed in the RABVs isolated from kudu, suggesting that these mutations may have occurred due to the adaptation of the virus to the host. Evidence from this study strongly argues for the maintenance of an independent RABV cycle in kudu, separate from the jackal cycles in Namibia. Future studies will be needed to determine the potential significance of the genomic regions identified as unique to kudu RABVs and to determine whether host specific regions occur in RABV and other lyssaviruses. The important implications of the phenomenon of the independent maintenance of RABV among kudu are that the control and eradication of a rabies cycle in this, and other species, will pose unique and unchartered challenges. From a continental perspective it is important to note that endemic canine rabies remains of primary concern and Namibia and other countries of the southern African region are no exception. Eventual effective control of rabies in kudu and jackal is likely to be subject to an effective control program for rabies in dogs.

## Supporting Information

Table S1 **Primers used for full RABV genome sequencing.**
(DOCX)

Table S2 **RABV full genomes used in phylogenetic and mutational analysis.**
(DOCX)
