# Supplementary material for: Complete Genome and Molecular Epidemiological Data Infer the Maintenance of Rabies among Kudu (Tragelaphus strepsiceros) in Namibia
Source: PLoS One. 2013 Mar 20;8(3):e58739. doi: 10.1371/journal.pone.0058739 (PMC3604114; doi:10.1371/journal.pone.0058739)
Supplement: Table S1 — Primers used for full RABV genome sequencing. (DOCX) [file pone.0058739.s001.docx]

**Table S1: Primers used for full RABV genome sequencing**

| **Primer Name** | **Primer Sequence (5’-3’)** | **Annealing Site** |
| --- | --- | --- |
| Pan-Lyssa-1F flap | AAT AAA TCA TAA TAC GCT TAA C | 1-9 |
| Pan-Lyssa-1.1F | TAC GCT TAA CRA CMA RAH CA | 1-19 |
| Pan-Lyssa-1581F | GAY HTK GAR ATG GCN GAD GAR AC | 1551-1584 |
| Pan-Lyssa-2191R | GGR AAY TTR TAY TTY TTN GAR AAR CTY TC | 2132-2160 |
| Pan-Lyssa-2682F | CCN CCN CCN GAR TAY GTB CC | 2595-2614 |
| Pan-Lyssa-2935R | CGT CKT AYY TTR TAN ACC CAR TTC A | 2824-2848 |
| Pan-Lyssa-3082F | GDN TNT GGT GYA TNA ACA YGA | 2995-3015 |
| Pan-Lyssa-3723R | GTG GTR GTN ACR TAM CCN ACR AAG TT | 3591-3616 |
| Pan-Lyssa-4059F | CAN ANN AGY ANA GGG AAG ADA GC | 3951-3973 |
| Pan-Lyssa-4472R | TTG TAR TGD GCA TCD GCY TCC AT | 4341-4363 |
| Pan-Lyssa-6417F | TAY ATG AAY GCN YTN GAY TGG GA | 5964-5986 |
| Pan-Lyssa-6607R | AGC ATN AGN GTR TAG TTY CTG TC | 6132-6154 |
| Pan-Lyssa-7222R | TCN CAN AYA TGT TTN GGD GGC CA | 6747-6769 |
| Pan-Lyssa-7107F | AGN TGG GGN TTT GAH AAR TAY TC | 6654-6676 |
| Pan-Lyssa-7561R | TAN ARY CTN ARA TTC CAN GAC AT | 7086-7108 |
| Pan-Lyssa-7620F | ACY ATG CAN GAC AAY YTG AAC AA | \| 7167-7189 \| \| --- \| |
| Pan-Lyssa-7744R | TGR TTG TTC CAY TTY TCR TAG TC | 7269-7291 |
| Pan-Lyssa-8280F | CCN GAR TCN AAR AGA TGG GC | 7827-7846 |
| Pan-Lyssa-8662R | CTN ANC CAD ATC TCT CTC CAG AA | 8187-8209 |
| Pan-Lyssa-8830F | TNT ATG AHG ARG TNG ACA AGG T | 8377-8398 |
| Pan-Lyssa-9034R | TYC TNA TNG THC GAG AGT TTT GDA T | 8556-8580 |
| Pan-Lyssa-9147F | TGY TCN KCN GAG AGR GCA GA | 8694-8713 |
| Pan-Lyssa-9216F | CCT CAC CCN TCN GAG ATG TT | 8763-8782 |
| Pan-Lyssa-9491R | GAN TYT CTN GHN ATG AAC CAG TTD AT | \| 9012-9037 \| \| --- \| \|  \| |
| Pan-Lyssa-9695R | GTN ARN TCN GAC ATN GTR TCT GT | 9219-9241 |
| Pan-Lyssa-10175F | TGA CAN GAA TGA CAN AYA TCA AYA T | 9721-9745 |
| Pan-Lyssa-10472R | CTY CTR AAG TCN GAR AAK ATC CA | 9996-10018 |
| Pan-Lyssa-10675F | ACN AGR TGG GTN GAT CAA GAR GT | 10221-10243 |
| Pan-Lyssa-11081R | GAN GCC ATN AGR TCA TTB ACC TC | 10605-10627 |
| Pan-Lyssa-11146F | ATN GAY BTT GAN TCN ATC TGG GA | 10692-10714 |
| Pan-Lyssa-11603R | CGN GCY CTH WGC ATC TCA CT | 11130-11149 |
| Pan-Lyssa-11656F | AAT CCN TAY AAT GAR ATG ATH ATA AC | 11202-11227 |
| Pan-Lyssa-11897R | GTD CTN CAR CAD ATR TTG AAG TG | 11421-11443 |
| Pan-Lyssa-12077F | TGA GTY TVT CNT CTC ACT GGA T | 11623-11644 |
| Pan-Lyssa-12407R-flap | AAT AAA TCA TAA ACG CTT AAC AAA | 11921-11932 |
| R13-JW12 | ATG TAA CAC CYC TAC AAT G | 55-73* |
| R14-RV-N-196F | GAT CCT GAT GAY GTA TGT TCC TA | 266-288* |
| Pan-Lyssa-Pyro-biot. 1 | TCC AAT TNG CAC ACA TTT TGT G | \| 662-641^ \| \| --- \| \|  \| |
| Pan-Lyssa-Pyro-biot. 2 | TCC ART TAG CGC ACA TYT TAT G | 662-641^ |
| Pan-Lyssa-Pyro-biot. 3 | TCC AGT TGG CRC ACA TCT TRT G | 662-641^ |
| RAB-JACKAL-100R | TAT TGA CTT TGA ATA CAA TCT TGT CG | 3’ RACE |
| RAB-JACKAL-150R | CTT TGA TAG CAG GGT ACT TGT AC | 3’ RACE |
| RAB-JACKAL-12235F | TAT TAG ACT ACA GCT GCT TGT AAA C | 5’ RACE |
| RAB-JACKAL-12265F | CTG CAC GTC CCA AGA CTT TTG | 5’ RACE |

N= any nucleotide; R = Purine; M = A/C; H = A/C/T; Y = Pyrimidine; V = A/G/C; D = A/G/T; W = A/T; B = G/C/T; K = G/T. All primer annealing sites are in relation to the Pasteur Virus strain (M13215) with the exception of primers marked * that correspond to the SADB19 (M31046) strain and ^ that correspond to CVS (GQ918139)
