# Supplementary material for: Complete Genome and Molecular Epidemiological Data Infer the Maintenance of Rabies among Kudu (Tragelaphus strepsiceros) in Namibia
Source: PLoS One. 2013 Mar 20;8(3):e58739. doi: 10.1371/journal.pone.0058739 (PMC3604114; doi:10.1371/journal.pone.0058739)
Supplement: Table S2 — RABV full genomes used in phylogenetic and mutational analysis. (DOCX) [file pone.0058739.s002.docx]

**Table S2: RABV full genomes used in phylogenetic and mutational analysis**

| **GenBank Accession Number** | **Sample Name** | **Country** | **Year** | **Host/Details** |
| --- | --- | --- | --- | --- |
| FJ577895.1 | Flury-LEP-C | China | 2008 | Vaccine strain |
| GU358653.1 | GX4 | China | 1994 | Dog |
| AB569299.1 | H-08-1320 | Sri Lanka | 2008 | Human |
| AB085828.1 | HEP-Flury | Japan | 2002 | Vaccine strain |
| EU643590.1 | HN10 | China | 2006 | Human |
| GU345747.1 | J | China | 1986 | Human |
| GU647092.1 | JX08-45 | China | 2008 | Chinese ferret badger |
| DQ875050.1 | MRV | China | 2006 | Mouse |
| AB128149.1 | Ni-CE | Japan | 2003 | Vaccine strain |
| AB044824.1 | Nishigahara | Japan | 2000 | Vaccine strain |
| EF437215.1 | NNV-RAB-H | India | 2007 | Human |
| DQ099525.1 | PM1503 | Germany | 2005 | Vaccine strain |
| EU182347.1 | RB/E3-15-5 | China | 2007 | Vaccine strain |
| EU182346.1 | RB/E3-15 | China | 2007 | Vaccine strain |
| EU311738.1 | RRV ON-99-2 | Canada | 1999 | Raccoon |
| EF542830.1 | RV-97 | Russia | 2007 | Vaccine strain |
| EF206717.1 | SAD1-3670 var 1 | Germany | 2007 | Vaccine strain derived from SAD |
| EF206718.1 | SAD1-3670 var 2 | Germany | 2007 | Vaccine strain derived from SAD |
| EF206709.1 | SAD B19 (Fuchsoral) | Germany | 2007 | Vaccine strain derived from SAD |
| EF206720.1 | SAD Bern (Sanafox) | Germany | 2007 | Vaccine strain derived from SAD |
| EF206719.1 | SAG 2 | France | 2007 | Vaccine strain derived from SAD |
| AY956319.1 | serotype 1 | Germany | 2005 | Human ex India |
| GU345748.1 | SH06 | China | 2006 | Dog |
| AY705373.1 | SHBRV-18 | USA | 1984 | Bat |
| AF499686.2 | SRV9 | China | 2004 | Vaccine strain |
| EU293121.1 | 8743THA | Thailand | 2008 | Human |
| EU293111.1 | 8764THA | Thailand | 2008 | Human |
| FJ959397.1 | CTN-1 | China | source 1956 | Vaccine strain |
| HQ317918.1 | CTN-1-31 | China | 1956 | Vaccine strain |
| EF564174.1 | CTN181 | China | source 1956 | Vaccine strain |
| GU345746.1 | CQ92 | China | 1992 | Dog |
| FJ712196.1 | F04 | China | 2008 | Chinese ferret badger |
| FJ712195.1 | F02 | China | 2008 | Chinese ferret badger |
| EU549783.1 | BD06 | China | 2008 | Dog |
| FJ712194.1 | D02 | China | 2008 | Dog |
| FJ712193.1 | D01 | China | 2008 | Dog |
| FJ866836.1 | FJ009 | China | 2008 | Dog |
| AB362483.1 | BR-Pfx1 | Brazil | 2009 | Hoary fox |
| GU565703.1 | Flury-LEP | China | 1948 | Vaccine strain |
| HM535790.1 | CVS-N2c | USA | 2010 | Challenge virus standard |
| GQ918139.1 | CVS-11 | France | 2009 | Challenge virus standard |
| GU565704.1 | Flury-HEP | China | 1948 | Vaccine strain |
| DQ875051.1 | DRV | China | 2006 | Deer |
| HQ450385.1 | DRV-AH08 | China | 2008 | Dog |
| FJ866835.1 | FJ008 | China | 2008 | Dog |
| EU293115.1 | 9147FRA | France | 1991 | Fox |
| HQ450386.1 | DRV-Mexico | Mexico | 2010 | Dog |
| EU293116.1 | 9704ARG | Argentina | 1997 | Bat |
| EU293113.1 | 9001FRA | Guyana | 1990 | Dog |
